# Synergizing strengths of four trauma-focused treatments in a highly intensive integrated program for posttraumatic stress disorder: rationale and two case descriptions

**DOI:** 10.3389/fpsyg.2026.1932004

**Published:** 2026-09-30

**Authors:** Mirjam J. Nijdam, Berthold P. R. Gersons, Ad de Jongh, Ruud A. Jongedijk, Agnes van Minnen, Tim R. Wind

**Affiliations:** 1 ARQ Centrum'45, ARQ National Psychotrauma Center, Oegstgeest/Diemen, Netherlands; 2 Department of Psychiatry, Amsterdam Public Health and Neuroscience, Amsterdam University Medical Centers, Amsterdam, Netherlands; 3 Research Department, Psychotrauma Expertise Center (PSYTREC), Bilthoven, Netherlands; 4 Academic Centre for Dentistry Amsterdam (ACTA), University of Amsterdam and VU University, Amsterdam, Netherlands; 5 School of Psychology, Queen's University, Belfast, Northern Ireland; 6 Institute of Health and Society, University of Worcester, Worcester, United Kingdom; 7 Behavioral Science Institute (BSI), Radboud University, Nijmegen, Netherlands

**Keywords:** brief eclectic psychotherapy for PTSD, commonalities, eye movement desensitization and reprocessing, massed treatment, narrative exposure therapy, posttraumatic stress disorder, prolonged exposure, trauma-focused psychotherapy

## Abstract

**Background:**

Various evidence-based trauma-focused psychotherapies for posttraumatic stress disorder (PTSD) are available and several commonalities have been identified. Yet, these trauma-focused psychotherapies also have important differences. This raises the question whether several trauma-focused methods can be combined in one treatment format. Thus far, systematic efforts to combine multiple trauma-focused treatment methods have been limited.

**Objective:**

To describe the rationale, structure, and application of a highly intensive treatment format integrating four trauma-focused psychotherapies, illustrated by two case presentations. The study was descriptive and was not designed to evaluate clinical effectiveness.

**Method:**

Description of a structured treatment format that integrates prolonged exposure (PE), eye movement desensitization and reprocessing therapy (EMDR), brief eclectic psychotherapy for PTSD (BEPP) and narrative exposure therapy (NET) in a highly intensive dose. Sessions were performed twice a day over the course of 1 week. This format was conceptualized in the context of a documentary series on an online ongoing education platform for therapists.

**Results:**

A combat veteran with PTSD and a survivor of long-term physical and sexual abuse with PTSD completed the 1-week program. Their treatment courses illustrate how the components of PE, EMDR therapy, BEPP, and NET can be organized and applied within a single intensive format. In non-standardized interviews conducted for the accompanying documentary, both participants described different functions and perceived contributions of the 4 treatment approaches.

**Conclusions:**

The case descriptions demonstrate that the proposed integration of four trauma-focused approaches could be applied as planned in two individual treatment trajectories of patients with severe PTSD. Patients' reflections were not collected or analyzed as formal qualitative data and cannot be used to establish treatment effectiveness, safety, or general feasibility of the treatment format. Prospective studies incorporating standardized outcome assessments, systematic adverse event monitoring, and formal qualitative evaluations are required to systematically investigate the treatment program.

## 1 Introduction

Several psychotherapeutic approaches have been developed to help alleviate the symptoms of posttraumatic stress disorder (PTSD; Bisson and Olff, 2021). In the past decades the field of trauma-focused interventions has progressed rapidly, providing patients with PTSD with opportunities to choose from an array of interventions that now have the predicate “evidence-based.” Many of these interventions were originally developed for specific traumatized populations. Prolonged exposure (PE; Foa et al., 2019) was initially developed for rape victims. Repeated exposure to traumatic memories and related stimuli is applied with the aim of emotional processing and decreasing fear and avoidance behavior in daily activities. Exposure may also promote inhibitory learning, whereby new associations are formed between previously feared stimuli and the absence of the expected aversive outcome (Craske et al., 2022). Narrative exposure therapy (NET; Schauer et al., 2011) was originally conceptualized with war and torture survivors from post-conflict areas in mind. With simple means such as a rope, stones, and flowers, a lifeline is laid out on which the most important life events are placed. Exposure to the traumatic events and attention for the positive events enables processing and is aimed at embedding them in autobiographical memory, resulting in a testimony. Brief eclectic psychotherapy for PTSD (BEPP; Gersons et al., 2015) was developed for police officers involved in shooting incidents. Exposure to a core traumatic event primarily addresses feelings of helplessness and grief, whereas anger is expressed in writing assignments. This is followed by reflection on the meaning and impact of the trauma in the context of someone's life. Indications for these three interventions broadened in clinical practice over time, which led to scientific evaluation of the treatment methods in other or more general populations of patients with PTSD, including people suffering from the repercussions of childhood abuse and military-related traumas. Eye movement desensitization and reprocessing therapy (EMDR) was based on the coincidental finding that eye movements reduced anxiety related to traumatic events, and a variety of tasks taxing patients' working memory have been applied since to process traumatic memories with the associated negative cognitions (Shapiro, 2018; Günter and Bodner, 2008; Lee and Cuijpers, 2013; De Jongh et al., 2024). Both trauma memory activation and working memory taxation are distinguishable components of EMDR therapy.

The abovementioned four treatment methods for PTSD are most commonly applied in trauma care settings in the Netherlands and have been proven efficacious in randomized controlled trials. Effect sizes of various forms of trauma-focused psychotherapy such as PE and EMDR therapy are roughly in the same range (e.g., Lewis et al., 2020), providing no specific indicators as to which treatment should be applied first. No consistent, strong predictors of treatment outcome have been found either for specific interventions (Barawi et al., 2020). National and international guidelines do not present a hierarchy of interventions based on theoretical grounds, although some make distinctions based on the strength of the evidence they reviewed in making recommendations for specific interventions over others (Akwa, 2020; International Society for Traumatic Stress Studies, 2019; American Psychological Association, 2025; National Institute for Health and Care Excellence, 2018).

International pioneers of empirically supported trauma-focused treatment have acknowledged the commonalities in their approaches (Schnyder et al., 2015). Common factors they identified were: psychoeducation; imaginal exposure; emotion regulation and coping skills; cognitive processing, restructuring and/or meaning-making; emotional processing; and memory processes. Looking at the four trauma-focused approaches described above, we see exposure and cognitive processing as key overlapping processes. Exposure can take the form of recounting the traumatic event in detail, imagining the most fearful or distressing parts of the traumatic event, or confronting triggers associated with the event. Cognitive processing may include challenging negative thoughts and beliefs about the world and self after a traumatic event, or exploring the meaning and impact of the event on the client's life. The many commonalities that were identified may facilitate the merge of various trauma-focused treatment protocols into a single comprehensive protocol. Apart from these commonalities, each treatment also has its own strengths and areas of focus.

Since several years, a common approach in trauma care is to offer highly intensive, 1 to 2-week programs to treat PTSD with trauma-focused techniques like PE, EMDR, or NET, which have shown to lead to significant reductions in PTSD symptom severity (e.g., Zepeda Méndez et al., 2025; Voorendonk et al., 2020; Strijk et al., 2025). Many of these highly intensive programs already combine PE and EMDR therapy within the same program. Based on the commonalities described by Schnyder et al. (2015), further integration can be envisioned by adding components originating from other trauma-focused treatment methods. Such a comprehensive synthesized program applied in a highly intensive format could potentially lead to a greater proportion of patients drawing benefit from treatment in the future, because 39% of patients do not respond to conventional psychotherapies for PTSD (Semmlinger et al., 2024). Furthermore, systematic review and meta-analytic evidence showed that intensive trauma treatment formats yield secondary benefits such as lower dropout rates (Sciarrino et al., 2020; Leithner et al., 2026) and one randomized controlled trial showed greater cost-effectiveness of intensive PE than regular weekly PE (Kullberg et al., 2023).

The objective of this paper is to provide a rationale and descriptive proof-of-concept for a treatment format that aims to integrate four trauma-focused psychotherapies: PE, EMDR, NET, and BEPP, and is offered in a highly intensive form. This integrated program was originally conceptualized as a documentary series in the context of an online educational platform for therapists (“The Trauma Tapes”; www.psyflix.net). By integrating multiple trauma-focused approaches, the program aimed to offer diverse pathways for accessing and processing traumatic memories. This may especially be relevant in case of complex trauma histories and symptoms, where single-method approaches may be insufficient (Nijdam et al., 2023). The program was developed to explore whether distinct procedures from four established trauma-focused therapies could be combined coherently within a single intensive treatment format. Whether such an integrative approach improves clinical outcomes compared with single-method trauma-focused treatment remains an empirical question.

In this paper we first focus on a theoretical description of the four trauma-focused psychotherapeutic methods that were integrated into the program. Second, we describe the integrative trauma-focused treatment format. Third, we describe two cases and their treatment courses as shown in the documentary. We conclude this paper by discussing the approach and implications in terms of research and clinical practice.

## 2 Theoretical background of the four trauma-focused psychotherapies

Below, we provide a succinct description of the four trauma-focused treatments EMDR, PE, NET and BEPP.

### 2.1 EMDR therapy

EMDR therapy is a relatively short protocolized treatment procedure that aims to attenuate (‘desensitize') memories of distressing events (Shapiro, 2018) as part of PTSD and other trauma-related disorders; (De Jongh et al., 2024). The protocol comprises eight procedural steps (“phases”). Phase I consists of history taking and case formulation, resulting in a treatment plan, including an initial map of a patient's potentially relevant trauma memories. Phase II involves preparing the patient for trauma work, which includes appropriate psychoeducation related to the effects of trauma and EMDR therapy. Phases III to VII consist of the activation and reprocessing of the traumatic memory by asking the patient to retrieve the memory and to concentrate on the combination of the presently held most disturbing image of the memory, a self-referencing dysfunctional belief, and its emotional and somatic components. The clinician then instructs the patient to concentrate on these elements of memory while simultaneously performing another task, most commonly following the therapist's hand with the eyes. The role of the therapist is mainly guiding the patient to focus on the internal experiences and the thought associations that tend to arise. After each set of eye movements, the patient is instructed to report any emotional, cognitive, somatic, and imagistic experiences or changes and then is encouraged to “go with” whatever freely comes up in awareness. Eventually, the memory reaches a point where internal subjective disturbances about the memory are perceived as neutral (indexed as a subjective unit of disturbance—SUDS- of zero), and adaptive and positive beliefs are rated as solidly believable on a validity of cognition (VOC) scale. Phase VII, dedicated to ending the session, prepares the patient for the interim between sessions. Subsequent sessions start with Phase VIII, which consists of reevaluation and integration and is ostensibly a vital link in the continuity of care.

A distinctive component of EMDR therapy is the use of a dual-attention task, most commonly rapid eye movements, while the traumatic memory is held in mind. Although early accounts questioned whether eye movements contributed effects beyond memory activation, subsequent dismantling studies and meta-analytic findings have demonstrated medium-sized additional effects of eye movements on the processing of emotional memories as compared to control conditions without eye movements (Lee and Cuijpers, 2013). Experimental research further indicates that eye movements and other cognitively demanding tasks reduce the vividness and emotionality of recalled memories by competing for limited working memory resources (Günter and Bodner, 2008; De Jongh et al., 2024). The available evidence therefore supports both trauma memory activation and working memory taxation as part of the working mechanism of EMDR.

### 2.2 Prolonged exposure

Prolonged exposure (PE) is a trauma-focused treatment consisting of three main components: psycho-education, imaginal exposure and *in vivo* exposure (Foa et al., 2019). During psycho-education, the main focus is on the explanation of how avoidance behavior and dysfunctional thoughts hinder processing the traumatic memories. During imaginal exposure, patients are confronted with memories of the traumatic event(s). Patients are instructed to describe in detail the most difficult and fearful parts of the traumatic memory that are typically avoided. To enhance the vividness of the memories during recounting, the patient has closed eyes and tells the memory in the present tense as if it happens here and now. To promote emotional processing, the feelings and thoughts evoked by imaginal exposure (for instance, feelings of guilt and shame) are discussed after the exposure exercise. In addition to imaginal exposure, exposure *in vivo* techniques are used for optimal activation of the fear network. *In vivo* exposure takes place in a safe context. Patients are confronted with feared trauma-related situations, places, people, and stimuli that are being avoided in daily life. *In vivo* exposure helps break the cycle of avoidance, enabling patients to challenge and violate their feared outcomes by experience. To enhance generalization, patients also engage in exposure homework exercises between sessions, both imaginal (for instance, listening to the audio recording of the imaginal exposure session) and *in vivo* (for instance, visiting the place where the trauma took place).

PE is theoretically based on the emotional processing theory (Foa and McLean, 2016), that posits that two conditions have to be met to successfully treat posttraumatic symptoms; first, the fear network that has been developed after the trauma containing all trauma-related stimuli has to be optimally activated, and second new, dysfunctional cognitions have to be replaced by more functional cognitions. During and after the exposure exercises, patients experience that their dysfunctional cognitions (for instance that the world is dangerous or that they are incompetent) are challenged. In addition, they learn that the harm that they expected when approaching trauma-related stimuli does not occur, and their harm expectancies are violated. These learning experiences and changes in the fear network eventually lead to fear extinction. In line with emotional processing theory, changes in fear and cognition seem to be the underlying mechanisms of exposure therapy (Cooper et al., 2017; Brown et al., 2019).

### 2.3 Narrative exposure therapy

Narrative Exposure Therapy (NET) is a treatment approach especially suited for PTSD resulting from multiple traumatic experiences (Schauer et al., 2011). NET is a time-limited therapy that usually varies from four to 16 sessions. The most essential elements of NET are the creation of the lifeline of the patient's most impactful life events and then working through these events on the lifeline by performing narrative exposure therapy in chronological order. A written testimony is made for the patient during treatment. More concretely, NET includes the following elements. Session one consists of diagnostic assessment and psycho-education. Session two involves creating and laying the lifeline, symbolized by a cord, followed by placing symbols of choice in chronological order along this lifeline. These symbols are successively *stones* (traumatic events), *flowers* (positive experiences), *candles* (moments of grief and loss), and *sticks* (aggressive acts). In session three the narration starts, beginning at birth and continuing to the first traumatic event. Every symbol is worked through by the narrative exposure technique. This technique has similarities but also important differences with the imaginal exposure technique in PE (Schauer et al., 2011). Instead of focusing on the most distressing parts of the traumatic memory, *narrative* exposure addresses the entire event, in chronological order from start to finish. The traumatic memory and explicit memories of its context are recalled, hypothesized to facilitate embedding in autobiographical memory (Conway and Pleydell-Pearce, 2000). NET also pays attention to the positive, resilient events in someone's life by discussing the flowers on the lifeline. After every session a short biographical report is written by the therapist. Every session starts with the rereading of the previous report, followed by the continuation of the chronological narration of subsequent parts of the lifeline and traumatic events. In the final session, the entire document is re-read, approved and signed, with the patient receiving the bundled report.

### 2.4 Brief eclectic psychotherapy for PTSD

Brief Eclectic Psychotherapy for PTSD (BEPP) is a 16-session manualized trauma-focused treatment comprised of five signature elements: *psychoeducation, imaginal exposure, writing assignments and mementos, meaning making and integration*, and a *farewell ritual* (Gersons et al., 2015). Effective elements originating from various therapeutic schools have been integrated in a logical sequence. The treatment starts with *psychoeducation* in the first session. Together with the partner or a close friend, the patient learns how the symptoms of PTSD are linked to the traumatic event(s), about mechanisms in the brain that are involved, and about the elements of the therapy. From session 2–6, *imaginal exposure* takes place. In the imaginal exposure a specific traumatic event is addressed in a very detailed way, part by part over the course of several sessions. This is done with closed eyes and in the present tense. The aim of the imaginal exposure in BEPP is to address feelings of helplessness and grief related to the trauma. This leads to a cathartic moment or the expression of “silent grief” in the sessions which is considered essential for trauma processing and successful BEPP treatment (Nijdam et al., 2013). During the subsequent exposure sessions, patients are asked to bring along *mementos*; objects that are literally or symbolically linked to the traumatic event. Parallel to the exposure sessions, patients start *writing letters* to express anger toward the person or organization they hold responsible for the traumatic event(s). Often these letters contain important themes for the next phase called *meaning making and integration*. In this phase lasting from session 7–16, patients identify important lessons they have learned from the traumatic event(s) in their lives. This helps them gain insight into the impact of the trauma on their life views and assumptions, and how to find a new equilibrium with the world they live in. The therapy ends with a *farewell ritual*, which is a symbolic way to leave the trauma and the disrupted period of life behind, and to make a fresh start.

## 3 Development of the program: the integration

The four trauma-focused treatments described above have demonstrated effectiveness in randomized clinical trials and formed the foundation for the design of the program. The aim of merging these approaches was to provide a balanced, logical sequence of interventions that would make sense to patients with PTSD related to multiple traumatic events. The current program was designed as a highly intensive treatment program, with 10 sessions taking place in the course of 1 week (twice daily sessions). The therapy sessions were given on a rotating basis by four different psychotherapists. Because the setup was new, the treatment program was carefully prepared and discussed by the team of therapists. In particular, the ethics for deviating from existing protocols, as well as the logical, theoretical, and practically substantiated order of the interventions/sessions, were carefully argued. Moreover, to guarantee high quality, the sessions were performed by leading trainers and therapists in the Netherlands regarding PE (AvM), EMDR (AdJ), BEPP (BPRG), and NET (RAJ), who had many mutual consultations during the week. The Trauma Tapes documentary series followed the entire treatment of the first two patients who underwent this program. The patients were extensively informed about the setup of the treatment program and its experimental character, the recordings, and the availability thereof on the online continuing education platform and agreed to these procedures and their case description in the current paper by providing written consent. Participants were selected based on basic eligibility criteria, including a clinical diagnosis of PTSD, willingness to engage in an intensive treatment format, and fluency in Dutch. Exclusion criteria applied at selection were severe dissociative symptoms, acute suicidality, and severe alcohol or substance use disorder. Participants were required to be available for full-time participation in a 5-day, week-long intensive program. Therapists were required to be trained in at least one of the trauma-focused methods as applied in the program and needed to schedule 15 min for transfer of information on progress before their session.

The order of the interventions was as described in Table 1. The first session consisted of psychoeducation and was taken from the BEPP protocol because it offers explanations of trauma and PTSD on multiple levels and in various domains of life. This session was characterized by thorough and detailed attention to the symptoms experienced by the patient, followed by a personalized explanation of the link between the symptoms and traumatic event(s). Its aim was to gain a deeper understanding of the breadth of the symptoms that belong to PTSD, to learn how PTSD impacts the brain and social relations, and to educate about how trauma therapy intends to decrease PTSD symptoms.

**Table 1:** Order of the trauma-focused psychotherapy interventions in the highly intensive integrated program.

Session	Intervention	Content
*Session 1*	BEPP Psychoeducation	Explanation of PTSD and trauma on emotional, cognitive, neurological, and social levels; linking personal symptoms to trauma; setting treatment expectations.
*Session 2*	NET Lifeline	Creation of a lifeline using symbolic items (stones, flowers, candles, sticks); identification of traumatic and positive events and periods across the lifespan.
*Session 3*	NET Exposure	Exposure to the traumatic event as a whole, in chronology, contextualizing fragmented trauma memories within the overall life story to reduce emotional disorganization.
*Session 4*	EMDR therapy	Targeting traumatic memory with high level of disturbance; focused on rapid reduction of emotional intensity.
*Session 5*	EMDR therapy	Continued reprocessing of traumatic memory; deepening emotional resolution and reduction in distress.
*Session 6*	NET Exposure	Additional integration of trauma memory; addressing unresolved aspects from earlier lifeline work.
*Session 7*	BEPP Exposure	Processing trauma involving intense emotions such as guilt or grief; emphasis on emotional expression and reflection.
*Session 8*	PE imaginal and *in vivo* exposure	Imaginal exposure to trauma memory and exposure *in vivo* to trauma-related stimuli for activation of the fear structure, reducing avoidance behavior, challenging feared outcomes.
*Session 9*	PE imaginal and *in vivo* exposure	Continued exposure work; activation of the fear structure, change of dysfunctional cognitions about self and world, and breaking cycle of avoidance behavior through repeated exposure.
*Session 10*	BEPP Meaning making and farewell ritual	Reflection on treatment gains, life lessons, and identity changes post-trauma; farewell ritual and emotional closure.

BEPP, Brief Eclectic Psychotherapy for PTSD; NET, Narrative Exposure Therapy; EMDR, Eye Movement Desensitization and Reprocessing; PE, Prolonged Exposure.

In the second session, a lifeline was created consistent with the NET protocol. The lifeline was laid out with a rope on the ground, along which stones which were placed for traumatic events, flowers for positive events, candles for events involving grief, and sticks for aggressive acts. The lifeline provided an overview of all the significant events in the patient's life within the context of the whole life story and showed that life was not all negative by pointing out all other life events and periods in life, including the positive. An important purpose of the lifeline in the current treatment was to select traumatic events that were targeted in the subsequent treatment approaches.

In session 3–7 several imaginal exposure approaches were applied that involved different methods of exposure, taken from NET, BEPP, PE, and EMDR protocols. Every one or two sessions a different type of imaginal exposure was applied and a different traumatic event was addressed. In principle, the order of the various approaches is interchangeable, but the approach was changed from session to session because of the different emphasis in the methods of imaginal exposure. The idea behind the alternation between treatment methods is that the type of exposure can be matched to the character of the traumatic event to draw optimal benefits. For EMDR therapy, particularly traumatic memories with the highest level of disturbance, from which the patient experienced intrusions, were selected. PE was selected when trauma-related avoidance was a prominent maintaining factor, either involving traumatic memories associated with intense fear, guilt or shame, or trauma-related situations and activities avoided in daily life. BEPP was chosen for the central traumatic experience that changed someone's life for the worst like a tipping point, which is often associated with grief, shame, guilt or anger. NET was chosen in case of a fragmented, disorganized traumatic memory which needed to be embedded in the autobiographical memory with the appropriate context in space and time. In this way, a separate order could be determined for each patient, depending on the chronology of the symbols on the lifeline, the level of distress, and specific emotions linked to a traumatic event. For an overview of the considerations when to apply which intervention we refer to Table 2.

**Table 2:** Proposed criteria to consider in the choice for a trauma-focused psychotherapy method in the highly intensive integrated program.

Intervention	Indication for the program	Objective
**EMDR therapy**	Selected especially for traumatic memories with the **highest level of disturbance**. These memories often cause intense intrusive images or flashbacks, or intense emotional and physical responses.	Resolution of distress related to the traumatic memory, operationalized as a Subjective Units of Distress rating of 0.
**Prolonged Exposure (PE)**	Selected for trauma memories that are **avoided** because they evoke intense emotions such as fear, guilt or shame, and **strongly maintain current avoidance behaviors or dysfunctional cognitions** that significantly interfere with daily functioning.	Activating the trauma-related fear structure and facilitating corrective learning through repeated imaginal and *in vivo* exposure, thereby modifying inaccurate or exaggerated threat-related perceptions and reducing avoidance.
**Brief Eclectic Psychotherapy for PTSD (BEPP)**	Selected for the **central traumatic experience** that represents a major turning point, (i.e., start or remarkable worsening of symptoms), or “tipping point” in the person's life (i.e., an event that motivates the person to seek help after a cumulation of traumatic events).	Processing the “hotspots” of the traumatic memory and giving words to the associated emotions such as grief, sadness, guilt and anger, with the aim of emotional expression rather than complete reduction of distress.
**Narrative Exposure Therapy (NET)**	Selected when the traumatic memory is **fragmented or disorganized, and lacks spatial and temporal context**, i.e., the person cannot remember the order in which things took place or only remembers (or speaks about) parts of the traumatic event.	Integration of the emotional, sensory, cognitive and bodily aspects of the traumatic memory (‘hot memory') with the factual, contextual memory (‘cold memory'), and recognizing this traumatic event in connection with the person's life narrative.

Sessions 8 and 9 consisted of imaginal exposure and *in vivo* exposure as applied in the PE protocol. Detailed imaginal exposure to the memories of the traumatic event took place in combination with *in vivo* exposure to safe but previously avoided stimuli without safety behavior applied by the patients. The objective was to allow the patients to experience that they could remember the traumatic event and related stimuli without the expected harm and that avoidance behavior would be countered. Homework assignments were included only following the PE sessions in line with the protocol, and involved tasks such as listening to session recordings and engaging in exposure *in vivo*.

The final 10^th^ session was dedicated to meaning-making and was drawn from the BEPP protocol. This session served to discuss the changes the traumatic events had caused in the patient's life in a broad sense and investigated if the person had become “wiser” in some way as a result of the traumatic events. The positive events defined on the lifeline were addressed here as well. This session further discussed the progress made in the treatment and the end of the treatment program.

In the current program, it was a deliberate choice to work with one session per traumatic memory. This structure allowed patients to experience multiple trauma-focused therapy modalities within a limited time frame. The definition of “sufficient processing” varied by treatment approach. For EMDR therapy, the target was a Subjective Units of Distress Scale (SUDS) rating of 0, indicating resolution of emotional distress. For NET, the goal was to integrate the emotional, sensory, cognitive and bodily aspects of the traumatic memory with the factual, contextual memories in the individual's life narrative. BEPP focused on processing the traumatic memory with the associated emotions such as grief, sadness, and anger, with the aim of emotional expression rather than complete tension reduction. For PE, the session centered on activating the fear structure and addressing dysfunctional beliefs linked to the trauma, with the goal of cognitive and emotional processing.

As part of the documentary production, participants were interviewed by members of the documentary team during and after the treatment week about their experiences of the sessions. These interviews were conducted for educational and documentary purposes rather than as part of a prespecified qualitative research design. No standardized interview guide was used for research purposes, the interviews were not subjected to systematic coding or qualitative analysis, and data saturation was not assessed. Accordingly, participant statements are presented only as illustrative personal reflections and should not be interpreted as qualitative research findings.

## 4 Case descriptions: first experiences

Two patients with multiple traumatic events in their life history, including early childhood trauma, were willing to try this highly intensive trauma treatment program. Both fulfilled the diagnostic criteria for PTSD according to DSM-5. The total score on the Clinician-Administered PTSD Scale (CAPS-5) was 44 for patient 1 and 42 for patient 2. As described above, the patients were informed about the nature of the treatment project, including the filming of the therapy sessions for the ongoing education platform.

### 4.1 Patient 1

The first patient was a woman in her early forties who had been abused in her childhood and later by her (now ex-) husband. She grew up in Eastern Europe with her mother and her grandmother was a motherly figure throughout her life. When she was 7, she came to the Netherlands to live with her father and stepmother. Her father started to physically abuse her and that was when she realized that her life had taken a turn for the worse. Physical and emotional abuse by father and stepmother and sexual abuse by father continued for 7 years. She was able to flee but the man who became the father of her children also physically abused her for years. After her divorce she found a loving boyfriend. Because she wanted to leave her traumas behind she responded to a call to participate in the program.

During the first (BEPP) session her partner participated in the psychoeducation session. They learned about the relationship between her symptoms and her traumatic experiences. She highlighted how much she appreciated the therapist listening to her story. In the second session (NET: the lifeline), it became clear to her that she experienced two turns for the worse in her life (moving in with her father at age 7 and falling in love with a man who mistreated her), but also that she could ultimately make life-saving decisions. She realized that her grandmother was very caring and loving, which gave her a firm foundation throughout life, as did her current boyfriend. In short, she could very well face some patterns in her life history and realized that she had both periods of great personal strength and periods of misery and despair. In the subsequent EMDR therapy and NET exposure sessions, she focused on processing the emotional impact of her abuse. In the BEPP exposure sessions, she processed the first time she realized that her life took a turn for the worse, namely, when her father had hit her with a whip. During the PE imaginal exposure sessions, it was difficult for her to recount the details of several rape memories vividly. She was afraid that she would be overwhelmed by emotions and would fall apart when doing so. During this exposure session, she was also confronted with trauma-related stimuli she avoided, such as sounds that reminded her of traumatic events (heavy breathing and footsteps on the stairs) and smells (smell of tobacco because her father smoked). Through repeated exposure she was able to face these triggers, and this emotional processing helped her realize that she could handle these reminders without being overwhelmed by fear or anxiety.

In the last (BEPP) session, she concluded that she wished that people would interfere when they would see child abuse as many people looked away in her past. She made it part of her life's mission to make people aware of child abuse, and she realized above all that she could now look at her traumatic memories from an adult's perspective without being overwhelmed by the emotions she had felt as a child. As a goodbye ritual, part of BEPP, she threw two stones (part of NET) in the water of a nearby ditch: one for her father and one for her ex-boyfriend to leave behind the disturbed periods of her life once and for all.

### 4.2 Patient 2

The second patient was a combat veteran in his early sixties, who had experienced multiple combat deployments. He had a 10-year history of mental health care and had been in trauma treatment focused on his missions for the Ministry of Defense before he enrolled in the Trauma Tapes. He grew up with parents who had both been imprisoned in Japanese camps during World War II in the former Dutch East Indies (now: Indonesia). This resulted in emotional neglect during his youth and it seemed like his parents were letting him relive their own camp experiences at times (e.g., he had to eat his own vomit or stand in the freezing cold as a young child). His father also used to physically hit him. At the end of adolescence, he chose to find structure in the military. His ability to neglect his feelings helped him carry out the most daunting missions, but early onwards, he lost men, had to take out enemies, and was witness to atrocities or fell victim to the latter.

In session one (BEPP) he could talk about his high expectations of the treatment but also about his doubts because he already suffered from PTSD for so long. In the second session, the NET lifeline helped him realize that he also had moments of joy throughout his life, whereas previously, it had seemed that life was always overshadowed by his fears and nightmares. “It cleaned up his attic,” as he would say, as the lifeline helped him to structure his traumatic memories, place events in chronological order, and recognize positive life events alongside the traumatic ones. In addition to flowers and stones, he also laid out a large number of important ‘sticks'. He noted that there were two episodes of violence and trauma in his life, which could be related: his father's aggressive beatings and his military deployment with many aggressive and life threatening incidents. In the BEPP exposure he described an extremely dangerous mission to free a prisoner. During this action the hand of a mate was severed. It was the same hand he had felt on his shoulder looking through the scope of his rifle when they were laying in the meadow for orientation at the start of the mission. During the BEPP exposure, he felt the sadness about the loss and how he felt responsible because he was in charge of the group. During the NET exposure sessions he gained more insight into the violent events in his life and the connection between his violent childhood experiences and his violent acts in adulthood. In addition, when the therapist read out the testimony report, his empathy for himself as a traumatized person grew. EMDR therapy focused on a memory of a fight with his father during his adolescence, in which he injured his father about which he felt guilty for the rest of his life. Another memory in an EMDR session also related to moral injury and was about taking revenge on an enemy combatant after he had been tortured by him earlier. In the PE imaginal exposure, he was asked to focus on feelings of fear during this traumatic experience. He was afraid that he would faint during the sessions and would be vulnerable to attacks. To prevent that, he admitted to wear several hidden instruments all over his body that could be used as a weapon, as if to be ready for a mission. For instance, he wore knives, melee weapons and tactical flashlights in his socks, shoes, cap, on his back, belt and in several pockets. During the PE exposure sessions, he was asked to put away every kind of instrument that could possibly be used as a weapon. During the exposure *in vivo*, the therapist strongly encouraged him to overcome his fear of touching doorknobs. In the army, he used be a specialist in explosives, where he installed and defused booby traps—often tied to doorknobs -, knowing very well how dangerous they could be on explosion. He would open doors only with a hand towel wrapped around the handle, as an extension of his hand, so that nothing could happen. He knew of course there were no booby traps in the building but this knowledge did not help him to overcome his fears that his hand would be torn off by a booby trap. During the exposure sessions, fear levels were rather high, but in the end he was able to open the door by himself which was very liberating. As a homework assignment, he was instructed to practice this new behavior (touching doorknobs and putting away the instruments he carried as safety behavior) between the exposure sessions in the hotel room where he was staying. In session 10 (BEPP) he reflected on what the week had meant to him and his perspective on life. He learned that his first strategy to meet unfamiliar people was to go forward and meet them just as in his missions (in line with the proverb “attack is the best defense”), and that he could change that. He was encouraged to find out who he was behind all the defenses he had learned to overcome a bit. He concluded that he could now say that he is good as a person and hesitantly looked forward to the future.

### 4.3 Personal evaluations

During the documentary interviews, both participants reflected positively on different components of the program. They described the NET lifeline as helping them organize their life histories and identify positive as well as traumatic experiences. They associated BEPP with reflection on the personal meaning of traumatic events and emotional expression, EMDR therapy with changes in the distress they experienced while focusing on selected memories, and PE with confronting avoided trauma-related situations and relinquishing safety behaviors. These statements represent the participants' subjective and retrospective impressions. Because they were not obtained through standardized outcome assessment or analyzed within a formal qualitative framework, they cannot be interpreted as evidence of symptom reduction, treatment effectiveness, or general feasibility.

## 5 Discussion

This paper describes a rationale and descriptive proof-of-concept for a highly intensive trauma treatment program of one week (10 therapy sessions integrating four distinct trauma-focused treatments given by four different psychotherapists), which was originally conceptualized as a documentary series for an ongoing education platform. To the best of our knowledge, this is the first attempt to merge these four trauma-focused approaches. A facilitator for the merge was the realization that trauma-focused treatments have a lot of important components in common which may even transcend their differences (Schnyder et al., 2015). Oftentimes though, separate therapeutic “schools,” scientific or financial interests to disseminate one specific approach over others prevent further thinking about integration. Different trauma therapies also have unique approaches and techniques, and in recent years attempts have been made to combine some of them. One example is the combination of PE and EMDR therapy in highly intensive trauma treatment programs which has shown to be highly effective in reducing PTSD symptomatology and complex forms of PTSD (e.g., Voorendonk et al., 2020). Such developments can be seen as inspired by the definition of “third wave” or “third generation” methods of cognitive behavioral treatments. This third wave provides space to encompass EMDR, NET and BEPP within a broader range of CBT interventions that are characterized by focusing more on the person's relationship to thought and emotion than on the content of behavioral or cognitive processes alone (Hayes and Hofmann, 2017). Combined “third wave” programs may offer patients the opportunity to work on their symptoms by means of varied approaches and see their symptomatology from a broader perspective. Whether this translates to augmented treatment effectiveness should be evaluated empirically.

In our program four trauma-focused approaches were chosen that are most commonly applied in the Netherlands to inform the current synthesis. This program was designed with patients in mind who suffer from PTSD related to multiple traumatic experiences. BEPP elements of psychoeducation and meaning making plus a farewell ritual sandwiched the other elements. The NET lifeline provided the overview of traumatic and resilient events and periods and facilitated the choice for events that would be addressed in the exposure sessions. EMDR, narrative exposure, and prolonged exposure sessions can subsequently be chosen interchangeably, depending on the nature of the traumatic event and the objective the therapist has in the session. Different treatment approaches may target different aspects of traumatic experiences and pursue different objectives during exposure sessions. During imaginal exposure in BEPP, the emphasis is primarily on grief or sadness that is linked to trauma memories, whereas in imaginal exposure in PE the focus is on trauma memories that are avoided because they evoke intense emotions such as fear, guilt or shame, and in NET on the elaboration of autobiographical memories and connections between sensory aspects and contextual information (the “hot” and “cold” memories). There are also differences in focus. NET focuses on placing the patient's entire history of traumatic events into the context of the person's course of life. Other trauma-focused psychotherapies concentrate more on addressing the most disturbing target images of the trauma memory (EMDR), repeatedly focusing on the “hotspots” of the trauma memory in great detail (PE) or zooming in on one “core” traumatic event (BEPP).

The two participants indicated that they appreciated and saw the additional value of the different therapeutic elements and there were no signs that they experienced discordance in the course of the program. The variety in approaches was also appreciated: within the therapy program, the patients were always addressed in different ways. These two case presentations show that the planned treatment components could be delivered within the proposed 1-week format in these two individuals; they do not establish the feasibility of the program more generally. An important limitation of the two case descriptions in this paper is that we did not have systematic post-assessments and follow-up assessments available on clinical outcomes of interest due to the context of the documentary production. The documentary production may also have influenced the participants in several ways, for instance in terms of giving socially desirable answers in the sessions or post-session interviews, or feeling inhibited to discuss negative aspects of the therapeutic alliance. This context along with the absence of quantitative or qualitative outcomes precludes statements about clinical effectiveness, safety or comparison of outcomes with established treatment protocols. Future iterations of the program should include standardized symptom assessments at multiple time points to enable more rigorous evaluation of treatment effects. If preliminary evidence is promising, the program should be evaluated in controlled clinical studies.

One can argue against our approach that the order of the different approaches is not completely fixed and could therefore encourage therapist drift during the application of the program or even within sessions. Although we give suggestions when to select a certain type of exposure or EMDR therapy, the choice for a certain approach could be substantiated further by more specific criteria and be informed by research. Patient factors such as the degree of dissociation, the presence of psychotic symptoms, and the severity of substance use disorders should also be considered in the case conceptualization. We also see it as very important that at least one therapist of a team who would provide such a program in the future would be adequately trained in all approaches, to make well-informed decisions when to apply a certain exposure strategy and write these down in a predetermined treatment plan so that those elements are not applied at random. Most of the current highly intensive programs work with therapist rotation, which has shown to lead to good ratings of the therapeutic relationship (Van Minnen et al., 2018). Therapist rotation may actually enhance adherence to the protocol and to pre-selected, exposure approaches for the traumatic events agreed on by the team. Just as with our two patients described above, it is important that there is careful transfer of crucial treatment content between the different therapists.

Another consideration regarding the current approach is that the additional value of a highly intensive integrative treatment program in comparison to regular trauma care, should be the subject of further study. The same is true for the frequency of the sessions, duration of therapy and the sequence of approaches in the sessions, as clinical outcomes may vary depending on which treatment is applied first (Van Minnen et al., 2020). Further research can also provide insight whether intensive integrated programs lead to secondary benefits such as less dropout and greater cost-effectiveness. A helpful approach to determine the effects of separate treatment components would be to perform frequent assessment of core symptoms or ecological momentary assessments throughout the program. Furthermore, while the current program focused on the integration of four trauma-focused therapies commonly used in Dutch clinical practice, other evidence-based treatments such as Cognitive Processing Therapy (CPT) and Cognitive Therapy for PTSD (CT-PTSD) were not included. We recommend that future iterations of programs like ours explore the feasibility and added benefit of incorporating CPT or CT-PTSD. Such additions could help identify optimal combinations of cognitive and experiential elements and contribute to enhanced treatment personalization.

In summary, this paper describes the rationale and structure of a one-week program integrating four trauma-focused psychotherapies and illustrates its application in two case presentations. The report demonstrates that the planned treatment components could be delivered within the proposed format in these two cases. Because no standardized quantitative outcomes, systematic qualitative data, or prospective safety data were collected, the present report does not provide evidence regarding clinical effectiveness, acceptability, safety, or added value relative to existing trauma-focused treatments. A prospective feasibility study followed by controlled clinical evaluations is required before the program can be recommended for routine clinical practice.

## Data Availability

The data analyzed in this study is subject to the following licenses/restrictions: Data are limited to the clinical interviews for the two case examples reported on in the manuscript. Due to the sensitive nature of these data, only the total scores are reported and the rest of the interviews are not publicly available. Requests to access these datasets should be directed to Mirjam J. Nijdam, m.mink@centrum45.nl.
